# The Prospective Beneficial Effects of Red Laser Exposure on *Lactocaseibacillus casei* Fermentation of Skim Milk

**DOI:** 10.3390/biology9090256

**Published:** 2020-08-31

**Authors:** Mahmoud S.M. Mohamed, Fouad M.F. Elshaghabee, Sulaiman Ali Alharbi, Ahmed El-Hussein

**Affiliations:** 1 Department of Botany and Microbiology, Faculty of Science, Cairo University, Giza 12613, Egypt; 2 Dairy Science Department, Faculty of Agriculture, Cairo University, Giza 12613, Egypt; elshaghabee@gmail.com; 3 Department of Botany and Microbiology, College of Science, King Saud University, P.O Box 2455, Riyadh 11451, Saudi Arabia; sharbi@ksu.edu.sa; 4 The National Institute of Laser Enhanced Science, Cairo University, Giza 12613, Egypt

**Keywords:** laser beam, *Lactobacillus casei*, skim milk, fermentation profile, probiotics

## Abstract

Probiotic lactic acid bacteria are crucial producers of fermented dairy products that are popular functional foods in many countries. The health benefits of probiotic bacteria are mainly attributed to their effective bioactive metabolites. The quality of fermented milk is mainly dependent on the bacterial strain used in the fermentation process. In this study, an innovative technique is used in order to enhance the activities of the probiotic bacteria, quality of fermented milk, and consequently the whole fermentation process. Red laser dosages, at the wavelength of 632.7 nm, were applied to the type strain *Lacticaseibacillus casei* NRRL-B-1922 before the fermentation of skim milk. The results revealed that the scavenging of 2,2-diphenyl-1-picryl-hydrazyl-hydrate (DPPH) radical and total antioxidant capacity were significantly increased from 21% in untreated control to 56% after bacterial laser irradiation of 12 J/cm^2^ dosage for 40 min. The antioxidant activity was found to be increased as the red laser dosage increased in a dose-response relationship. Additionally, the lactose fermentation in skim milk medium of 43.22 mg/mL initial concentration into organic acids was enhanced after *L. casei* irradiation and recorded 23.15 mg/mL compared to control group 28.35 mg/mL without bacterial pre-treatment. These results are correlated with increase of the β-Galactosidase activity, where the *L. casei* that has been exposed to 40 min of red laser exhibited the higher activity of a 0.37 unit/mL relative to the control 0.25 unit/mL. The assessment of this fermented milk after *L. casei* laser exposure for 10, 20, and 40 min indicates multiple biological effects, including assimilation of cholesterol as well as proteolytic and antibacterial activity. Our data on the exposure of *L. casei* to laser beam suggest promising application of red laser in the fermentation process of skim milk.

## Highlights

(1) Exposure of *Lactocaseibacillus casei* before fermentation process of skim milk enhance the antioxidant capacity of fermented milk. (2) *L. casei* proteolytic and β-galactosidase activities were significantly induced after the laser exposure dosage of a 12 J/cm^2^. (3) Increasing the red laser beam exposure time up to 40 min significantly enhanced the lactose fermentation profile of fermented skim milk. (4) The fermented skim milk level of cholesterol was significantly reduced after *L. casei* exposure to red laser beam compared to untreated bacterial control.

## 1. Introduction

Fermented milks which contain living microbial strains, namely probiotics, represent the first functional dairy products with a wide range of biological functions brought by the probiotic effective bioactive metabolites [1]. Several research groups investigated the health benefits of probiotics bacteria, mainly strains belonging to the genera *Lactobacillus* and *Bifidobacterium*, in fermented milk and dairy products. These benefits include among many anti-carcinogenic effect [2], anti-obesity, antioxidant effects [3,4], and an enhancement of the immune response [5].

*Lacticaseibacillus casei* formerly named as *Lactobacillus casei* have been reclassified recently based on the whole genome sequences analysis to novel genera, *Lacticaseibacillus* (the *L. casei* group) [6]. This new genus remains relatively heterogeneous and the strains belonging to this genus have a considerable economic importance as probiotics and starter cultures in dairy fermentations [6]. Some strains are well known to have a vital role in the ripening of different types of cheese, like Cheddar cheese [7,8]. The *L. casei* NRRL-B-1922 is recognized as safe by both the US Food and Drug Authority and by the European Food Safety Authority and many studies demonstrated its probiotic properties [9]. Different strains of *L. casei* possess many beneficial effects, e.g., *L. casei* 01 could assimilate cholesterol from broth media [10]. *L. casei* Shirota strain [11] and *L. casei* GMNL 263 [12] could reduce the plasma cholesterol level and attenuate non-alcoholic fatty liver disease in animal models. 

Recently, there are many biomedical applications that involve lasers; where these applications vary from diagnostic tools as spectroscopy to therapeutically approach like photodynamic therapy [13,14]. Increased level of visible laser radiation can induce adverse side effects via reactive oxygen species (ROS) where the intracellular scavenging system is imbalanced with the produced free radicals. However, lasers of low energies have been deployed for several years in a versatile array of medical applications that include wound healing and skeletal injuries. Low intensities of coherent laser radiations have unique characteristics that mimic sunlight and thus have beneficial effects on biological systems [15]. This approach is called photo biomodulation (PBM) where lasers with low energies are used for therapeutic indications with minimal thermal impacts. Red and near-infrared lasers are in specific line within the phototherapeutic window and thus have many clinical applications with a high safety margin compared to the UV and blue regions [16]. The latter are known for their ability to stimulate aging, hyperpigmentation and carcinogenesis even with extremely low doses varying from 0.1 to 100 J/cm^2^. PBM has been used extensively in the therapeutic approach of many disorders and diseases. Despite that the exact mechanism of action of PBM is not yet well understood, low laser doses can have altered actions by cellular signaling induction in different routes [17]. Non-photosensitizing cells absorb laser radiation mainly through intracellular photo receptors called chromophores. For microorganisms like *Escherichia coli*, cytochrome bd and bo_3_ complexes are found to be the key player in the biological alterations induced by light absorption. They are responsible for stimulating transduction processes which lead to intracellular signaling pathways, and hence enlarging the effect of the primary absorbed light signal [18]. The molecules that are recruited in transducing such signals are chemically reactive species as reactive oxygen, whereby they react with other biomolecules and cellular structures to change their functions and/or expressed genes. Moreover, PBM was found to be a promising research area in the medical field because of its huge and enormous capabilities. The metabolism and proliferation of many living organisms including microorganisms can be accelerated and enhanced by red laser light. Light in the red region of the visible spectrum has an important effect and can stimulate the activity of antioxidant enzymes. Such enzymes may include ceruloplasmin, superoxide dismutase and catalase. There are many factors that could affect the PBM efficiency and its biological significance such as the emission mode (continuous wave or pulsed), exposure time, laser fluency, monochromaticity, and directionality [19]. To date, there are no experimental data exploring the effect of red laser beam on the characteristics of lactic acid bacteria. Therefore, in this study, an innovative technique is used to enhance the fermentation and probiotic activities of the lactic acid bacteria type strain, *L. casei* NRRL-B-1922 using different doses of red laser beam. Consequently, such an improvement of the bacterial functional properties will be related directly to the fermented milk’s beneficial health characteristics that would surge its marketing target audience and economical importance.

## 2. Materials and Methods

### 2.1. Microorganisms

*L. casei* NRRL-B-1922 was gratefully provided by Northern Regional Research Laboratory (NRRL), Peoria, USA. *Staphylococcus aureus* EMCC 1351, *Bacillus subtilis* EMCCN 1152 and *Escherichia coli* ATCC 25922 were obtained from Egyptian Microbial Culture Collection (EMCC), Microbiological Resources Center (MIRCEN), Faculty of Agriculture, Ain Shams University, Cairo, Egypt.

### 2.2. Laser Exposure

**Figure 1:**
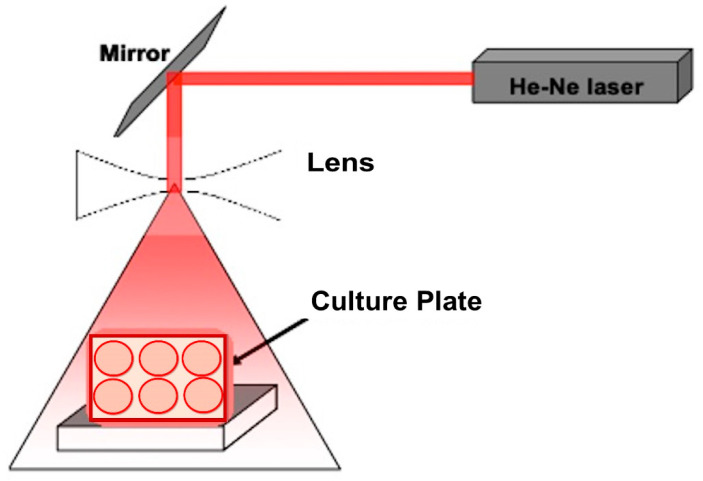
Experimental setup for culture exposure to red laser.

The laser exposure setup was based on utilizing a He-Ne laser at the wavelength of 632.7 nm (Photon, Al aboor, Egypt). The power of the used laser was 40 mW. A diverging mirror was used to reflect the laser light downwards into a telescopic system where the beam was expanded to cover the 6-well culture plate (SPL Life Science, Gyeonggi-do, South Korea) with a spot of 3.14 cm diameter with even and homogenous distribution throughout the plate as shown in Figure 1. A power meter (ThermoFisher Scientific, Waltham, MA, USA) was used to measure the laser power at different sites of the well plate. The laser doses that were employed on the *L. casei* cultures at the log phase were 3, 6, and 12 J/cm^2^ for 10, 20, and 40 min exposure time respectively. 

### 2.3. Bacterial Culture Cultivation

*L. casei* was cultivated using de Man, Rogosa and Sharpe (MRS) broth medium (Merck, Darmstadt, Germany) and incubated at 37 °C for 24 h. *E. coli*, *B. subtilis*, and *Staph. aureus* were cultivated in nutrient broth (Oxoid, Hampshire, UK) 37 °C for 24 h. 

### 2.4. Preparation of Fermented Skimmed Milk by L. casei NRRL-B-1922

Skimmed buffalo milk (0% fat, 9.32% total solids and 4.3% lactose) was heated at 90 °C for 10 min followed by cooling to 40 °C. The *L. casei* cultures were activated in MRS medium till the range of colony-forming units was 8.25–8.40 Log CFU/g. The skimmed milk was then inoculated with 2% (*v*/*v*) of activated *L. casei* cultures pre-treated with different laser doses as described in Section 2.2. Milk was fermented at 42 °C until pH reached 4.62 ± 0.05, then fermented milk was cooled to 15 °C in ice water bath and then stored at 4 °C till analysis. The control group was milk fermented by *L. casei* cultures without laser treatment whereas the negative control was sterilized skimmed milk. 

### 2.5. Preparation of Whey Fraction

The whey fraction of fermented milks was prepared as described by Virtanen et al. [3]. This can be summarized as follows. Bacteria-free fresh skimmed milk was used as a negative control. Fifteen mL of fermented milk free from non-hydrolyzed casein were obtained by dropping the pH 4.6 by using 1 N HCl. Then this mixture was centrifuged at 6000× *g* for 20 min at 5 °C and the supernatant was filtered (0.45 μm pore diameter). The filtrate was stored for further analysis.

### 2.6. Antioxidant Assay 

#### 2.6.1. DPPH Radical Scavenging Activity

Antioxidant activity of fermented skimmed milk based on the scavenging activity of the stable DPPH (2,2-diphenyl-1-picryl-hydrazyl-hydrate) free radical was determined by the method outlined by [20]. The mixture of 1:1 of 0.25 mM DPPH solution in 95% ethanol and whey fractions were mixed and left at room temperature for 30 min then 2 mL of deionized water were added, and the absorbance was measured by a spectrophotometer at wavelength 517 nm. The antioxidant activity was represented as percentage of DPPH scavenging activity.

#### 2.6.2. Determination of Total Antioxidant Capacity

The total antioxidant capacity of the fermented skimmed milk was assayed by the phosphor-molybdenum method as described before [21]. The method depends on the ability of whey fraction filtrate to reduce molybdenum VI to molybdenum V at acidic medium and hence the production of green complex of phosphate molybdenum V. Consequently, a spectrophotometer (Jenway 6305; Bibby Scientific Ltd., Staffordshire, UK) was used to measure the intensity of this green color at 695 nm, which indicates the antioxidant capacity as mg ascorbic acid equivalent per ml fermented milk. 

### 2.7. Fermentation Profile of Fermented Skimmed Milk

The profile of milk fermentation was assessed using high performance liquid chromatography (HPLC) as previously described before [22]. The procedure started by mixing 1 mL of filtered fermented milk with 10 μL Carrez I and 10 μL Carrez II (Sigma-Aldrich; St. Louis, MO, USA). The mixture was centrifuged at 14,000× *g* for 10 min at 4 °C then filtered (0.45 μm pore diameter) and the supernatant was stored till analysis at −20 °C. Directly before analysis, the samples were diluted 1:25 (*v*/*v*) with 0.0085 N sulfuric acid for assessment of the milk fermentation profile of lactose, lactate, and acetate.

### 2.8. β-Galactosidase Activity of L. casei Strain

The β-Galactosidase activity in fermented skimmed milk was performed as described by [23]. In brief, fermented milk was diluted with 50 mM phosphate buffer (1:10; *w*/*v*), then 200 µL lysozyme with a concentration of 50 mg/mL (Sigma-Aldrich; St. Louis, MO, USA) were added and mixed. After 30 min of incubation at room temperature, the mixture was centrifuged at 6000× *g* for 10 min at 4 °C. The supernatant was clarified by mixing 1 mL with 0.5 mL of Clarifying Reagent (Sigma-Aldrich; St. Louis, MO, USA) for 20 min. In order to measure the β-galactosidase activity, 1 mL of clear supernatant was mixed with 4 mL of 100 mM ortho nitrophenol-*β*-d-galactopyranoside dissolved in 100 mM phosphate buffer pH 7.0. The reaction mixture was incubated at 37 °C for 10 min in a thermomixer. The reaction was stopped by adding 2 mL of sodium carbonate 625 mM. The absorbance was determined at wavelength of 420 nm using a spectrophotometer against the reagent blank. The unit of β-Galactosidase (U) was defined as the amount of the enzyme that decreased 1 μmol of o-nitrophenol per min at 37 °C per 1 mL of fermented milk [24].

### 2.9. Cholesterol Assimilation by L. casei before and after Treatment

The cholesterol assimilation by *L. casei* with/without different treatments with laser was determined using MRS broth medium supplemented with water soluble cholesterol (Sigma-Aldrich; St. Louis, MO, USA) at final concentration of 2 mg/mL in addition to 0.2% sodium taurocholate (Merck; Darmstadt, Germany) as previously described by [10,25] where the supplemented medium was inoculated by a 2% activated *L. casei* culture and incubated for 18 h at 37 °C to mimic the intestinal conditions. The *L. casei* suspensions were centrifuged at 14,000 rpm at 4 °C for 10 min to collect the supernatant. A calorimetric method was used to investigate the cholesterol residues by measuring the absorbance at 550 nm [26].

### 2.10. Determination of Antibacterial Activity

The antibacterial activity of the prepared whey fractions was assessed by using the agar well diffusion method as instructed by the Clinical and Laboratory Standards Institute (CLSI) against selected microbial organisms [27]. Briefly, a single colony of overnight *E. coli*, *Staph. Aureus,* and *B. subtilis* cultures were used to prepare 0.5 MacFarland bacterial cultures (approximately 10^8^ CFU/mL). The plates of Müller-Hinton agar were inoculated with the individual tested bacterium and the wells of 5 mm diameter were filled with 100 μL whey fraction filtrate (0.45 μm pore diameter). After incubation for 24 h at 37 °C, the agar plates were examined to record any inhibition zones surrounding the wells. As a positive control, ampicillin (150 µg/mL) was used as standard antibacterial agent. The whey fraction of fermented milks, using *L. casei* without laser treatment, was used as negative control.

### 2.11. Determination of Proteolytic Activity

The degree of protein hydrolysis (DH) of fermented skimmed milk was measured using the o-phthaldialdehyde OPA assay. Donkor et al. (2007) established this method that can be outlined by mixing 0.75% trichloroacetic acid and whey fraction in 2:1 ratio and the mixture was filtered using membrane filter of 0.45-μm pore diameter [28]. The filtrate was incubated at room temperature with OPA for 4 min and the absorbance of the solution was measured at 340 nm as free amino groups by a spectrometer. The relative proteolytic activity was calculated relative to unfermented milk substrates.

### 2.12. Statistical Analysis

All the experimental assays were performed in triplicates independent experiments (*n* = 3) and analyzed using mean variance of ANOVA where *p*-value < 0.05 was set as the significant level. Origin Pro 8.3 was the software used throughout this study. 

## 3. Results and Discussion

### 3.1. Antioxidant Activities of Pre-Treated L. casei with Red Laser

**Figure 2:**
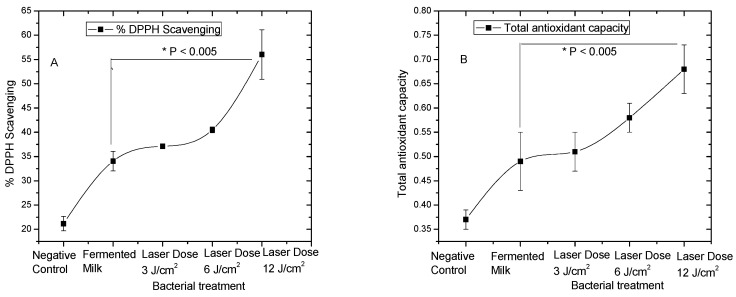
Effect of red laser irradiation on *L. casei* before skim milk fermentation as analyzed by (**A**): DPPH scavenging activity. The bars on the graph represent mean ± SD as percentage of radical scavenging activity of three independent experiments (*n* = 3). and (**B**): Total antioxidant capacity. The bars on the graph represent mean ± SD equivalent to mg ascorbic acid /mL fermented milk of three independent experiments (*n* = 3). * The difference was considered statistically significant (*p* < 0.05).

The total antioxidant capacity of the fermented milk was investigated by using scavenging DPPH radical and found to be the greatest at the group that has been irradiated for 40 min and with an irradiance of 12 J/cm^2^ (56% DPPH scavenging) as shown in Figure 2.

DPPH has been employed as a steady free radical in the investigation of peptides’ capabilities to hunt free radicals [29]. The control group that did not receive any light treatment displayed the minimum antioxidant capacity (21% DPPH scavenging), where the latter increased with increasing the light dose. The bacterial group that received the higher dose of 12 J/cm^2^ showed statistically significant increase in the DPPH scavenging activity and the total antioxidant capacity (*p* < 0.005) compared to the fermented milk without any laser treatment as shown in Figure 2A,B.

Several studies have reported that such antioxidant activity is dependent on the used bacterial strain, incubation temperature and duration of the incubation [30,31]. Many studies have demonstrated the effect of red and far red regions of the light spectrum on the antioxidant activities. Indeed, it was reported that catalase activity has been enhanced by increasing the photon energy of the laser excitation source. This enzyme plays a key role in the intracellular control of peroxide radicals. Low level laser irradiation (LLLI) was proven to change the catalytic activities of many enzymes [32,33].

### 3.2. L. casei Profile of Lactose Fermentation after Red Laser Exposure

**Figure 3:**
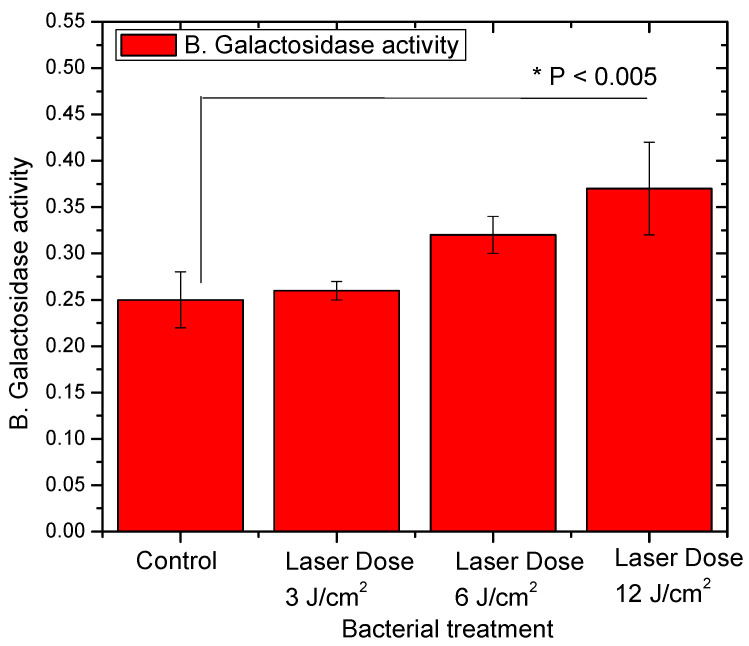
A Histogram showing the change of β-Galactosidase activity (Unit/mL) of *L. casei* without laser exposure (control) and after exposure to 3, 6 and 12 J/cm^2^ laser dosages. * The difference was considered statistically significant (*p* < 0.05).

The profile of lactose fermentation by the studied *L. casei* was found as well to be affected by the different red laser doses. The ability of this strain to convert lactose into lactic acid and acetic acid was studied with an initial lactose concentration in skimmed milk medium of 43.22 mg/mL. The lactic acid and the subsequent acetic acid concentrations have been shown to be the most with a red laser dose of 12 J/cm^2^ (6.12 and 0.92 mg/mL respectively). Such acetic acid concentrations are significantly higher than those of the control group (*p* < 0.005) as shown in Table 1. Based on these observations, one could assume a significant higher lactose fermentation capability of the studied *L. casei* strain. The same trend has been demonstrated in the activity of β-Galactosidase enzyme where the *L. casei* that has been exposed to 12 J/cm^2^ of red laser exhibited the higher activity of 0.37 unit/mL as shown in Figure 3. This increase of the β-Galactosidase activity is statistically significant (*p* < 0.005) when compared to that of the control group.

β-Galactosidase is an important enzyme of the hydrolyzation of milk and this process can be done before or during the milk fermentation [34]. The hydrolysis process done by β-Galactosidase is considered as one of the most important catalytic processes in the biotechnology and dairy industry. The impact of lactose hydrolysis on the specific characteristics of the fermented milk is mainly dependent on the starter and the used substrates [35]. Lactose hydrolysis can accelerate the fermentation process resulting in dairy products that can be used by lactose intolerant people as well as reducing the sugar content and hence the energy in the different dairy products. An interesting study in agreement with our results reported that blue as well as red light doses have increased the activity of β-galactosidase in wild type with salt stress soil when compared to unexposed bacteria (i.e., control group) [36].

### 3.3. Effect of Red Laser on Antibacterial Activities of L. casei

The antibacterial activity has been measured as a function of the diameter of the inhibition zone in the cultural plates. In agreement with the previous assays, the higher the laser dose, the more antibacterial activity achieved in the three tested bacterial strains, two from Gram-positive bacteria (*Staph. aureus* and *B. subtilis*) and *E. coli* as a Gram-negative bacterium. Moreover, laser dose of 12 J/cm^2^ has been found to significantly increase the antibacterial activity when compared to the untreated group of the *L. casei* filtrates against all the tested bacteria (*p* < 0.005 in case of *S. auerus* and *p* < 0.05 for *B. subtilis* and *E. coli*), as shown in Table 2, where the diameter of the starting zone was 5 mm. 

Blue light has been used extensively as an antimicrobial agent in several studies, despite that its mechanistic action is not clear yet. Red laser as well has been demonstrated to be toxic for many pathogens with a similar proposed mechanism to the blue light which is via porphyrin molecules. They have two absorption bands, one mainly in the blue and the other in the red region [37]. There could be different cellular photosensitizer potentials for light therapeutic modes. The porphyrins independent action mechanisms still need more research and studies. Bacteria with their variable cell wall structure and classifications comprise different optical characteristics that would influence light treatment modalities [38]. There should be an equilibrium and consideration between the quantum yield needed for killing a single pathogen and how deep can the used light penetrate through the tissue and its wavelength [39]. Kohli and Gupta have demonstrated that oxygen species production is induced by IR and visible light. This indicates the presence of appropriate chromophores in those prokaryotic cells that have not been specifically discovered yet [40]. On the other hand, the antibacterial effects of *L. casei* secondary metabolites after pre-treatment with red laser that have been observed on both Gram positive and Gram negative bacteria could be through the high concentration of organic acids or via the enhanced production of antimicrobial peptides, bacitracin antibiotics, and organic compound such as 2-pyrrolidone-5-carboxylic acid [10,41].

### 3.4. Red Laser Enhanced Cholesterol Assimilation and Proteolytic Activities of L. casei

*L. casei* can assimilate cholesterol and this ability was shown to be increased after dosing our strain with red light. The ability of assimilating cholesterol is found to be almost 41% in the bacterial group that has received laser dose of 12 J/cm^2^ as shown in Table 3. This higher percentage is statistically significant when compared to the unexposed *L. casei* (*p* < 0.05). 

Many researchers have noticed the decrease of the level of cholesterol in blood serum for men in certain African tribes after their ingestion of valuable quantities of *Lactobacillus* rich milk products. Such observation attracted a lot of attention towards the various and promising beneficial impacts of lactobacillus bacteria on fat metabolism in human and consequently on human health. Despite the numerous research works on different strains of bacteria, particularly *Lactobacilli* in the in vitro cholesterol reduction via their uptake of the fats into their cell membranes, complementary in vivo studies are far fewer to make such an effect in evident [42]. Tahri et al. revealed that the ability of the bacterial strain in assimilating cholesterol relies on their growth phase based on their findings that bacteria in the rest phase do not react with lipids [43]. Karu et al. performed a very interesting quantitative study a long time ago to demonstrate the photo-biostimulating effects of laser light of low intensity on different biological models including prokaryotic and eukaryotic cells. The study revealed the significant increase of the DNA synthesis and hence in the bio-stimulation of those models [44]. The stimulated growth of *Lactobacillus* bacteria by red light could be the justification of their enhanced ability of assimilating cholesterol. Another interesting study by Salama et al. 2012 revealed a stimulating effect of low laser intensities on the cholesterol degrading capacities of microorganisms [45]. 

The proteolytic activity of *L. casei* has shown to be increased after exposure to different laser doses before fermentation. The bacterial group that received laser dosed for 40 min showed the maximum proteolytic activity as of 6.3% as (Table 4) compared to the untreated bacterial group which expressed 5.1% proteolytic activity. This difference in the proteolytic activity between the two experimental groups is found to be statistically significant (*p* < 0.05).

Milk proteins are the main amino acid source for lactobacillus bacteria for their growth and development [46]. Genus *Lactobacillus* has many proteolytic enzymes that are involved in the degradation of such proteins into smaller peptides. Different strains of lactobacillus bacteria have different proteolytic capacities with complicated proteolytic structure and takes place intracellularly. Such strains can breakdown more than 40% of α_S1_-CN and β-CN peptide bonds that release more than hundred variable oligopeptides that are primarily released in the fermentation process of the milk. LLLT and what is recently known as PBM have many potential medicinal, agricultural and industrial applications [47]. Low laser doses with the correct wavelength are able to stimulate variable cellular signal pathways and enzymatic activities. The red region of the electromagnetic spectrum in particular can stimulate many anti-oxidant enzymatic activities, like catalase, ceruloplasmin, and superoxide dismutase [48]. Those effects could be vital for many biological systems through their stimulating actions on other proliferative, metabolic, and homeostatic processes [49,50,51]. 

## 4. Conclusions

In conclusion, the exposure of *L. casei* NRRL-B-1922 to laser doses 12 J/cm^2^ before skimmed milk fermentation exhibited a significant improvement of the antioxidant capacity, β-galactosidase, antimicrobial, and proteolytic activities. Simultaneously, it reduced cholesterol and lactose levels of fermented skimmed milk and thus enhanced the fermentation process of skimmed milk prepared with L. casei. However, other probiotic bacterial species should be tested individually with different red laser doses to ensure the same enhancement of fermentation process. The elucidation of the exact mechanisms of such activation requires further investigation for a better understanding and hence more applications. In large scale fermentation, delivering the light doses to the starter bacterial culture in a cost controlled and feasible way in fermentation plants will be a future challenge.

Following suitable procedures, this red laser pre-treatment method of probiotic bacteria can be used at the industrial scale to improve the quality of fermented milk and consequently the economic benefits.

## Figures and Tables

**Table 1:** Effect of red laser beam on lactose fermentation profile (mg/mL) by *L. casei*.

Treatments	Lactose Residues	Lactic Acid	Acetic Acid
Control	28.35 ± 0.51 a	4.82 ± 0.51 a	0.25 ± 0.06 a
3 J/cm^2^	28.15 ± 0.42 a	4.80 ± 0.48 a	0.24 ± 0.05 a
6 J/cm^2^	25.41 ± 0.37 b	5.70 ± 0.40 b	0.62 ± 0.03 b
12 J/cm^2^	23.15 ± 0.36 c	6.12 ± 0.71 c	0.92 ± 0.10 c

Initial lactose concentration in skim milk medium was 43.22 ± 0.62 mg/mL. The mean values sharing different letters indicate significant differences (*p* < 0.05) within the same parameter.

**Table 2:** Effect of red laser beam on antibacterial activity (diameter of inhibition zone) by *L. casei*.

Treatments	*Staph. aureus*	*B. subtilis*	*E. coli*
Control	10.20 ± 0.60 a	9.30 ± 0.80 a	7.50 ± 0.20 ab
3 J/cm^2^	10.30 ± 0.40 ab	9.40 ± 0.35 ab	7.45 ± 0.60 a
6 J/cm^2^	11.50 ± 0.50 cd	10.20 ± 0.52 abc	8.40 ± 0.30 bc
12 J/cm^2^	12.10 ± 0.30 d	11.00 ± 0.40 cd	9.15 ± 0.70 cd
Ampicillin *	14.73 ± 0.40 e	15.93 ± 0.61 e	12.70 ± 0.52 e

Inhibition zone diameter was measured and expressed in millimeter ± Standard deviation, * Ampicillin 150 µg/mL. The mean values with different letters indicate significant differences (*p* < 0.05) within the same bacterial species, while mean values sharing at least one common letter are not significantly different.

**Table 3:** Effect of red laser beam on cholesterol assimilation by *L. casei* before (control) and after irradiation by laser beam.

Treatments	% Cholesterol Assimilation
Control	38.12 ± 1.20 a
3 J/cm^2^	38.42 ± 0.65 ab
6 J/cm^2^	39.25 ± 1.35 abc
12 J/cm^2^	41.15 ± 0.83 c

The different letters indicate significant differences (*p* < 0.05), while mean values sharing at least one common letter are not significantly different.

**Table 4:** Effect of red laser beam on proteolytic activity by *L. casei* before (control) and after exposure to laser beam.

Treatments	% Proteolytic Activity
Control	5.10 ± 0.40 a
3 J/cm^2^	5.20 ± 0.25 ab
6 J/cm^2^	5.62 ± 0.30 abc
12 J/cm^2^	6.30 ± 0.55 c

The different letters indicate significant differences (*p* < 0.05), while mean values sharing at least one common letter are not significantly different.
